# Angels and Demons: Using Behavioral Types in a Real-Effort Moral Dilemma to Identify Expert Traits

**DOI:** 10.3389/fpsyg.2016.01464

**Published:** 2016-10-25

**Authors:** Hernán D. Bejarano, Ellen P. Green, Stephen J. Rassenti

**Affiliations:** ^1^Department of Economics, Center of Economic Research and TeachingAguascalientes, Mexico; ^2^Economic Science Institute, Chapman UniversityOrange, CA, USA; ^3^School for the Science of Health Care Delivery, Arizona State UniversityPhoenix, AZ, USA

**Keywords:** cognitive capabilities, personality, preferences, real effort, abstract effort, moral dilemma, experiment, survey

## Abstract

In this article, we explore how independently reported measures of subjects' cognitive capabilities, preferences, and sociodemographic characteristics relate to their behavior in a real-effort moral dilemma experiment. To do this, we use a unique dataset, the Chapman Preferences and Characteristics Instrument Set (CPCIS), which contains over 30 standardized measures of preferences and characteristics. We find that simple correlation analysis provides an incomplete picture of how individual measures relate to behavior. In contrast, clustering subjects into groups based on observed behavior in the real-effort task reveals important systematic differences in individual characteristics across groups. However, while we find more differences, these differences are not systematic and difficult to interpret. These results indicate a need for more comprehensive theory explaining how combinations of different individual characteristics impact behavior is needed.

## Introduction

Mainstream economic theory routinely assumes that individuals have stable, consistent preferences that at least partly determine their behavior and revealed preferences (Samuelson, 1948; Stigler and Becker, 1977). Behavioral and experimental economists have explored the validity of that assumption, and phenomena like preference reversals, endowment effects, framing, and the Ellsberg paradox imply that individuals lack stable, consistent preferences.

Most lab experiments attempt to induce consistent preferences using conditional rewards based on Smith's (1976) Induced Value Theory. In these experiments, failure to observe the behavior implied by the induced preferences leads researchers to question the narrow self-interest hypothesis and search for alternative theories. This process has contributed to a deeper understanding of preferences by examining how experimental designs and subject characteristics affect behavior (Frank and Glass, 1991; Becker, 2013). For example, experimental results imply that subjects are partially motivated by fairness (Rabin, 1993), equality (Bolton and Ockenfels, 2006), ambiguity aversion (Fox and Tversky, 1995), and identity (Akerlof and Kranton, 2000).

We argue that even with substantial improvements over the past decades in our understanding of how individual characteristics correlate with individual actions, several key questions remain: Are there systematic differences among individuals? For example, do variations in individual characteristics matter? If so, which characteristics influence behavior? Do actions reveal more than psychological indicators of behavioral types? Furthermore, little is known about how the answer to these questions depends on the elicitation method.

There are two prevalent approaches used to try to answer these questions: (1) surveying with primary experiments; and (2) adding secondary experimental tasks. In the first approach, researchers use questionnaires either before or after the primary experimental task. For example, several authors have explored how psychological characteristics influence economic behavior using this method—e.g., personality traits (Almlund et al., 2011; Ferguson et al., 2011); emotions (Pixley, 2002); and sentiments (Smith and Wilson, 2013). Corgnet et al. (2015) found that reflective individuals, as measured by the Cognitive Reflection Test (CRT), exhibited more consistently mildly altruistic actions in a lab experiment. Frederick (2005) and Burks et al. (2009) found that cognitive capabilities related to time and risk preferences. Other researchers investigated the interaction between personality traits and risk and time preferences (Rustichini et al., 2012). Researchers have also linked experimental behavior to the results of testing such for IQ (Oechssler et al., 2009; Brañas-Garza et al., 2012, 2015), social intelligence (Takagishi et al., 2010), and personality (Almlund et al., 2011; Rustichini et al., 2012). However, the findings are not consistent with one another (Ben-Ner et al., 2007; Eckel and Grossman, 2008; Borghans et al., 2009; Hirsh and Peterson, 2009; Oechssler et al., 2009; DeAngelo et al., 2015).

The alternative approach is to add secondary experiments that are designed to measure preferences or characteristics. Researchers use these measures to determine the relationship between a subject's actions in the primary experiment and their individual preferences or characteristics. Examples of this practice are the use of the Dictator Game, the Trust Game and Risk and Time Preference experiments as complements to primary experiments. Unfortunately, correlations between behavior in the primary and secondary experiments have not been consistent. For example, while characteristics such as risk preferences have accompanied behavior in games such as repeated prisoner's dilemmas and beauty-contest games (Boone et al., 1999; Sabater-Grande and Georgantzis, 2002; Goeree et al., 2003; Brocklebank et al., 2011; Lönnqvist et al., 2011; Kagel and McGee, 2014), the same characteristics sometimes failed to correlate (Aycinena et al., 2014). Another approach has found that in prisoner dilemma games, there are interesting evolutionary explanations for the existence of different types (Congleton and Vanberg, 2001).

In this article, we alter these approaches to address the inconsistencies described above. First, we utilize individual-level subject data collected on different occasions. That is, our measures of individual characteristics and preferences were collected in different experimental sessions from our primary experiment. We argue that, while difficult, using data collected from different experimental sessions implies that subjects are less likely to be influenced by portfolio and wealth effects across tasks. Secondly, we leverage a large dataset with over 30 measures of individual characteristics and preferences, the Chapman Preferences and Characteristics Instrument Set (CPCIS). These include measures of several types such as: personality traits, preferences, strategic behavior in simple games and the socio demographics of our experimental subjects. Furthermore, the CPCIS was not designed or implemented by us, so it reduces the potential presence of any experimental demand effect. More specifically, the CPCIS not only measures characteristics that we hypothesize to influence the behavior in our primary experimental task, but also a large set of variables which a priori should not influence actions in it.

Our primary experiment, based on Green (2014), presents experimental subjects with a novel real-effort experiment with a distinct moral dilemma. Subjects in this experiment representing experts are asked to provide proofreading services to another group of subjects (customers). The quality of the expert's edits affects the customer, positively if the edits are done properly and negatively if they are done incorrectly. However, the quality of edits has no impact on the expert's personal earnings. Therefore, the experts face a moral dilemma between maximizing personal earnings and providing benefits to their customer.

Behavior in moral dilemmas is hypothesized to be influenced not only by subjects' induced payoff function and preferences for monetary rewards, but also in other-regarding preferences, subject's cognitive capabilities, values and personality traits (Bowles, 1998; Fehr and Fischbacher, 2003). Therefore, we combine observed behavior from our primary experiment, a real-effort moral dilemma task, with the individuals' measures of the CPCIS to see how individual characteristics relate to an individual's actions.

Our results provide several new insights concerning experiments with a moral dilemma. Initially, we find that simple correlational analysis provides an incomplete explanation of how individual measures relate to behavior. Both measures of preferences and other individual characteristics fail to consistently correlate with actions in our main experimental task. For example, measures of individual preferences (i.e., risk aversion, loss aversion, and time preferences) are not correlated with observed actions in the primary experiment. In contrast, some measures of strategic preferences, intelligence, and personality are significantly correlated with behavior. However, in spite of the inconsistency in correlation across individual preferences and behavior, that fact that some measures do correlate is of note. When a subject's preferences are characterized by a combination of factors such as personality, cognitive capabilities, and intelligence, as in our primary experiment, predictions of behavior become uncertain. For instance, subjects with high measures of intelligence should produce higher outcomes for their customers, whereas those same individuals may have varying levels of altruism also influencing their behavior and, thereby, theoretical predictions.

This leads us to explore individuals by behavioral groups, also known as *clusters*. Clusters are identified using the action variables “total edits” and “total incorrect edits.” Cluster analysis based on these two variables allows us to distinguish between subjects who edited a lot with a high percentage of incorrect edits (the Demons) and subjects who edited sparsely with a high percentage of incorrect edits, as well as those who edited few with a high percentage of correct edits (Angels). Behavioral group members exhibited systematic differences in their individual characteristics. We found significant differences among behavioral groups that could not be detected using simple correlation analysis, suggesting that the effect of psychological, cognitive, and demographic differences on behavior in trials with our moral dilemma experiment is nonlinear. These results indicate a need for more comprehensive theory explaining how different individual characteristics work together.

## Experimental design and individual data

### Experimental design

The primary experimental design was introduced by Green (2014). The experimental design and data analyzed here are from Bejarano et al. (2016). Green's original experiment was designed to explore behavior between an expert and customer where the expert is presented with a moral dilemma. Experts are asked to provide proofreading services for a panel of customers. The quality of the expert's proofreading services affects the customer's wellbeing (in the form of monetary payment); however, the customer's wellbeing has no impact on the expert's personal earnings. Therefore, the experts are faced with a tradeoff between maximizing personal earnings and providing benefits for their customer.

The interaction between the expert and the customer took place in two phases with one group of subjects playing the role of the customer (Phase I) and another group playing the role of the expert (Phase II). In Phase I, customers were given 50 min to proofread 10 essays. Each essay had 10 typographical or spelling errors (e.g., misuse of “their” for “there” or “write” for “right”). Customers were initially endowed with $25; however, for each error they were unable to find, they lost $0.25. Phase I was designed to create customer demand for the proofreading services provided in Phase II of the experiment.

In Phase II, experts were presented with a panel of 40 customer-edited essays collected in phase I. These essays contained a total of 125 errors. To create the expert subjects, errors were highlighted when presented to the “experts.” In addition to the 125 errors that were highlighted, another 250 sections of text were highlighted to create a potential for over-editing.

There were three possible payment schemes for the expert: *fee-for-service, capitation*, or *salary*. Under *fee-for-service*, experts were paid $0.20 per individual field of text edited. Under *salary*, experts were paid a flat rate of $25 to participate in the experiment. Under *capitation*, experts were paid $0.625 for each essay in which they edited at least one highlighted section of the text. The expert's edits directly impacted the payoff of their customer. For each incorrect edit, the experts made to the text, customers lost $0.15 and for each correct edit, customers are reimbursed $0.05.

Each payment scheme presented a different moral dilemma; that is, strategies to maximize personal earnings or minimize effort varied across payment schemes. Under *fee-for-service*, experts faced a tradeoff between maximizing the number of edits and the quality of each edit for their customers. Under *salary*, experts faced a tradeoff between leaving the experiments early (minimizing effort) and providing services for their customers.^1^ Experts paid under *capitation* faced a tradeoff between the number of customers and the quality of edits for each customer.

In addition to varying the payment scheme, we also varied the expert's ability to select among the payment schemes. Our experiment included two treatments. Under the first, *self-selection*, experts could choose among the three payment schemes. Under the second, random assignment, experts were randomly assigned to one of the three payment systems: *fee-for-service, capitation*, or *salary*.

In Green (2014), subjects were randomly assigned to these payment schemes. Consistent with experts randomly assigned in the present analysis, experts in the *fee-for-service* treatment provided significantly more services than those in either the *capitation* or *salary* treatments. This difference was caused by a significant increase in the number of unnecessary edits to the essays provided by the experts, resulting in a much lower quality of service under the *fee-for-service* option compared to the *salary* or *capitation* payment schemes.

### The chapman preferences and characteristics instrument set (CPCIS)

Starting in September 2015, the ESI required all subjects to complete the CPCIS prior to participating in ESI experiments. This instrument set required about 90 min of a subject's time and was run independently of any other experiment, at a time convenient to the subject. The data collected by this instrument set consisted of standardized measures of preferences and individual characteristics gleaned from a series of classic simple experiments and questionnaires.

Measures are calculated for and sorted into five characteristic categories: individual preferences, strategic preferences, intelligence, personality tasks, and demographic characteristics. Individual preferences measured in the CPCIS include time preferences, loss aversion, and risk aversion. Strategic preferences include *trust* (adapted from Berg et al., 1995), *fairness* (adapted from Güth et al., 1982), and *altruism* (adapted from Kahneman et al., 1986).

Intelligence is measured using classic psychology measures from Raven, the CRT, and Wonderlic. Additionally, subjects are asked to complete a simple adding task, once with incentives for correctness and once with none. Social intelligence is measured using The Reading the Mind in The Eyes task. Finally, subjects provided self-reported measures of intelligence via their SAT and ACT scores, as well as their GPA. Personality was measured using the Big Five personality test. Demographic variables included age, gender, volunteer hours per week, work hours per week, number of siblings, number of older siblings, and finally, religiosity.

Although, the tests used are somewhat arbitrary and controversial, the results predict behavior in traditional experimental games and are consistent with several behavioral and experimental-economics studies that attempt to elicit relevant preferences. The goal of the CPCIS is to provide a panel dataset that includes the personality indicators most used by experimental economists, with indicators used by psychologists, sociologists, anthropologists, and other social scientists.

In order to integrate several traditional tasks within the same instrument set, tasks within CPCIS such as Raven, The Reading the Mind in The Eyes task and Wonderlic (Test, 1992) were truncated. Specifically, the CPCIS contained the odd-numbered questions from the last three series of matrices within the Raven test (Jaeggi et al., 2010), one that has also been used by Corgnet et al. (2015). Our Big Five questionnaire is based on the 44 items described by John et al. (2008). Conversely, we used an extended version of the CRT (Frederick, 2005). While the original task from Frederick (2005) has three questions, our task has seven questions (Toplak et al., 2011).

In addition to traditional games that elicit several types of other-regarding preferences, the CPCIS includes an instrument that elicits social preferences a la Bartling et al. (2009), hereafter referred to as the *BFMS task*. This task has been used to study preferences of subjects who self-select into competitive tasks (Bartling et al., 2009), as well as the relationship between cognitive capabilities and other-regarding preferences (Corgnet et al., 2015). In our experiment, we combine features of these two applications. Selection into a payment scheme is not based on competitiveness but tradeoffs between the desire to reimburse others and to maximize personal earnings. Therefore, we argue that selection into the different treatments could be related to social preferences elicited by the BFMS. In the following paragraphs, we briefly describe the BFMS that the students in the CPCIS faced^2^.

The BFMS instrument is a series of binary choices with different allocations for the decision maker and a randomly matched partner (Table 1). Each choice presents an egalitarian alternative and a non-egalitarian alternative. In our modified BFMS instrument, subjects have to make six choices. Of these six choices, three present subjects with a choice between an egalitarian alternative and another non-egalitarian division earnings, which is at least as good or favorable for herself but detrimental for the matched partner (choices BFMS1, BFMS2, and BFMS5). In contrast, two of the other three binary choices presented to the subject ask her to choose between the egalitarian alternative and a division that is as least as favorable for the matched partner but less than or equal for the decision maker (BFMS3, BFMS6). Finally, BFMS4 is welfare-improving or increases overall earnings but by a greater amount for the matched partner.

**Table 1:** **Bartling binary choice task**.

**Binary choice variable name**	**Egalitarian alternative A**	**Non-egalitarian alternative B**
BFMS1	Both subjects earn $10	Decision-Maker earns $10, Matched Partner $6
BFMS2	Both subjects earn $10	Decision-Maker earns $16, Matched Partner $4
BFMS3	Both subjects earn $10	Decision-Maker earns $10, Matched Partner $18
BFMS4	Both subjects earn $10	Decision-Maker earns, $11 Matched Partner $19
BFMS5	Both subjects earn $10	Decision-Maker earns, $12 Matched Partner $4
BFMS6	Both subjects earn $10	Decision-Maker earns $8, Matched Partner $16

In the CPCIS, after all of the subjects made their decisions, two of the individuals were randomly selected to have their choices determine the earnings for this task. Models describing behavior observed in the BFMS task vary across publications. Fehr and Schmidt (1999) presented a two-parameter α, β model, where α represents aversion to disadvantageous inequality, Behindness Aversion, and β aversion to advantageous inequality, Aheadness Aversion. Fehr and Schmidt (1999) assumed that α > β > 0. In contrast, Corgnet et al. (2015) related these parameters to envy and compassion and did not impose any assumption on them. The authors summarized five motivations that could make subjects select one alternative over the other. These include self-interest, altruism, egalitarianism, spitefulness, and inequality-seeking. The authors also said that individuals could have a combination of these motives while choosing among alternatives. In order to organize BFMS choices in a way useful for our analysis, we further simplified the choices within three types of preferences. Decision makers who chose alternative A more often across all six choices demonstrated egalitarian preferences. Decision makers who chose to allocate larger earnings to their matched partner than to themselves (alternative A in BFMS3, BFMS4, and BFMS5) at no cost or a small cost to their own earnings, were considered altruistic or averse to being ahead of their partner. Finally, decision makers who were more likely to choose option A in BFMS1, BFMS2, and BFMS5 were considered Spiteful. These individuals could also be considered as having demonstrated aversion to being behind their partner.

Based on these notions, we constructed three variables based on the BFMS choices for each individual. Each individual could choose between zero and six egalitarian alternatives (Egalitarianism). Also, they could choose between zero and three beneficial alternatives (Selfishness) or detrimental alternatives (Altruism). These three variables elaborate on the theory of other-regarding preferences and improve our understanding of how a subject's choices under this instrument relate to their actions in our moral dilemma experiment.

We do not claim that the measures obtained by these truncated tasks mirror those obtained by the original tests, but for the purpose of our analysis, we determine the extent to which these measures are correlated with the experimental actions.

## Theoretical relationship among CPCIS variables

In this section, we analyze the theoretical implications of expert preferences and characteristics. Two experimental-design features are important for our analysis. First, an expert in the self-selection treatment likely reveals something about her personal preferences in her selection of payment systems. Experts who are randomly assigned to their payment scheme will be the average of the general student population, rather than the conditional averages for the subject types that prefer a particular payment scheme. We will distinguish between these two groups in our predictions.

Second, the quality of the expert's proofreading directly impacted the customer's payment. But it had no impact on the expert's personal earnings. In the choice of a payment scheme, all experts in the self-selection treatment faced the same tradeoff, or moral dilemma, between choosing the payment scheme that would maximize personal earnings or one that would limit their maximum earnings. Therefore, selecting a payment scheme may reveal something about subjects' characteristics.

The following *ceteris paribus* predictions highlight the expected relationship between each individual characteristics and behavior in the primary experiment. However, we note that individuals do not differ from each other in *ceteris paribus* ways; therefore theoretical implications are unlikely to describe the expected differences in behavior among any two given subjects.

### Predicted behavior with homo economicus preferences

The predicted behavior varies with assumptions about expert preferences that are not induced. However, there are simple predictions for the outcomes of these experiments if we assume subjects prefer to be purely self-interested (*homo economicus)*. If careful editing requires bearing a real-effort or cognitive cost, a *homo economicus* expert assigned to the *salary* scheme will exert no effort and conduct no edits. A *homo economicus* expert randomly assigned to the *capitation* scheme should exert the minimum effort and only conduct one edit per essay. A *homo economicus* expert assigned to *fee-for-service* should maximize the number of edits with minimum effort and make both necessary and unnecessary edits. Furthermore, in the *selection* treatment, *homo economicus* would select *fee-for-service* 100% of the time, because under that scheme, experts can earn three times more than the maximum earnings possible under *salary* or *capitation*.

However, the experimental evidence presented in Green (2014) and Bejarano et al. (2016) demonstrates that subjects deviated from income-maximizing strategies. These results suggest that subject preferences were more complex than those of *homo economicus*. This leads us to investigate what role additional preferences might be in play in order to modify our assumptions regarding the effects of the payoff schemes on actions.

### Predicted behavior with other preferences and choice-relevant characteristics

The experimental design has some implications concerning the relevance of other personal characteristics as well. For example, risk aversion, loss aversion, and time preferences should not affect behavior. Subjects earnings do not depend on the correctness of their editing but only on their payment system and their decision to edit or not. Payments are deterministic. Therefore, subjects do not face risks of the usual kind. Similarly, the effect of choice on earnings is almost immediate; hence, time preferences should not influence choices.

On the other hand, a subject's actions in Phase II have an impact on the earnings of subjects who participated on Phase I. Therefore, we expect that measures of what might be regarded as social preferences should affect behavior. For example, differences in the extent of altruism is likely to affect behavior, as has been found in Dictator, Trust, Ultimatum Game, and Prisoner's Dilemma experiments. We expect measures of altruism to be positively correlated with efforts to help subjects in Phase I. Error rates should fall under *fee-for-service*, and more time (and care) should be spent editing under salary and capitation.

The three variables described above (Egalitarianism, Selfishness, Altruism) have an intrinsic relationship with what we expect to uncover with the selection and related actions in our experiment. We expect that those demonstrating Selfishness through these measures will prioritize their earnings over their customers'. Hence, these subjects will likely select *fee-for-service* and perform a larger number of edits rather than maximize their incomes, even at the expense of their customer. In contrast, those individuals that prioritize the earnings of their matched partners will likely choose *salary* and only attempt to conduct beneficial edits for the customers, even at a cognitive and time cost to themselves.

In contrast to the preference measures, predictions regarding Intelligence and demographic variables are not clear. Little is known regarding how actions in our experiment will be influenced by a subject's demographic characteristics. We also have variables that reflect Numeracy, Academic, and IQ Intelligence. To the best of our knowledge, this is the first time that researchers aimed to explore how these measures correlate with performance on incentivized linguistic tasks that affect third parties.

### Cognitive capabilities and personality

In this section, we clarify the implications that cognitive capabilities and personality traits could have on the behavior observed in our primary experiment given their indirect relationship with strategic preferences. In a novel study, Corgnet et al. (2015) found that Chapman students with a more reflective nature were less likely than intuitive individuals to be associated with egalitarian *and* spiteful motives. The authors named the behavior of those with scores above median CRT as mildly altruistic. Given that we have access to the same subject database with the same measures of cognitive capability (CRT) and preferences for egalitarianism or spitefulness (Bartling et al., 2009), we might expect also that subjects with higher CRTs would show some type of characteristic behavior. However, it is not clear what exactly would comprise mildly altruistic behavior in our experiment. The moral dilemma at hand implies that for each treatment, experts face a different tradeoff between self-interest and customer welfare. We expect subjects with higher CRT scores to be more likely to balance this tradeoff differently in the various treatments examined because they are more likely to reflect on the cost of the tradeoff at stake.

In an attempt to relate personality traits to preferences measures, Rustichini et al. (2012) used a dataset with 1000 truck drivers. They measured the truck drivers' Big Five traits, time preference, risk aversion, truck accidents, job persistence, credit score, and body mass index (BMI). The authors found that personality traits had stronger predictive power than time preferences or risk aversion for truck accidents, job persistence, credit score, and BMI. However, the authors argue that both economic and psychological theories are needed to understand truck-driver behavior.

Big Five personality traits are also likely to help explain differences in the behavior of experts among treatments and payment systems. Unfortunately, the Big Five factors are not orthogonal. Although, qualitative predictions can often be made for individual factors, a person's particular vector of factors often includes factors with the opposite effects on the behavior of interest. For example, *openness* is associated with curiosity and a higher willingness to explore. Therefore, relatively open individuals might be more likely to conduct a larger number of edits and to spend more time on them.

Conscientiousness is associated with being dependable and disciplined. In our experiment, experts have a mission. In their mission, they know that they could affect the earnings of their customers. Higher conscientiousness is likely to be correlated positively with measures of correct edits. Agreeableness is associated with higher cooperation against the exploitation of others (Andersen et al., 2006). We expect that subjects with higher agreeableness should conduct more correct edits to increase the earnings of customers. These three dispositions, therefore, tend to induce better outcomes for the customers.

Higher extroversion is associated with higher sensitivity to rewards. In this case, the perceived nature of the reward matters. Subjects with a higher extroversion measure (maintaining the degree of preferences for others' welfare) may be driven by monetary rewards. In that case, they will be more likely to choose *fee-for-service* and to conduct unnecessary edits. However, if they perceive their reward to be correlated with the benefits of their customers, extroverts will take greater account of such effects than introverts.

Finally, neuroticism appears to be the factor that is not likely to influence the behavior of subjects in a clearly predictive way. Because the experimental environment is set up to isolate subjects from situations where moods, anxiety, and depression play a significant role, we do not expect to find any significant correlation between neuroticism and behavior.

## Experiment, data, and analysis

The experiments were conducted in the ESI laboratory and conference rooms at Chapman University between May 2014 and May 2016. Experimental subjects were recruited from the ESI database of more than 2000 students. Phase I was conducted either in the ESI laboratory or the ESI conference room. Phase II was conducted in the ESI's computer laboratories. Printed instructions were provided for the students to read on their own for 10 min. At the end of the 10 min, the experimental coordinator read the instructions out loud. Subjects were not able to start the experiment until they satisfactorily completed a quiz.

Many of these subjects were also recruited to participate in the CPCIS by a different recruitment email on a previous date convenient to the subject's schedule. The CPCIS sessions were implemented in the same laboratory but had no formal connection to any other experiments being conducted at ESI. The local Institutional Review Board (IRB) approved both studies. In both studies, participants received a show-up fee of 7 USD plus additional incentive payments earned by their behavior in the session.

In the primary experiment, there was a total of 20 undergraduates (customers) recruited in Phase I and 228 undergraduates (experts) recruited in Phase II. In Phase II, which was dedicated to experts performing editing services, 105 subjects were randomly assigned to their payment scheme, and 125 selected their payment scheme. Of the subjects in Phase II, 161 had completed the CPCIS; 115 of those were in the *self-selection* treatment and the other 46 were randomly assigned to one of the three payment schemes. We focus our analysis below on the behavior of those 161 subjects who participated in the primary experiment and had undertaken the CPCIS. The primary experiment lasted an average of 1 h and 15 min, and completion of the CPCIC instrument required an average of 1 h and 35 min.

In the primary experiment, expert subjects could edit correctly or incorrectly. We will focus our analysis on six experimental actions: total edits, total incorrect edits, percentage wrong, net impact on the customer earnings, expert earnings, and total editing time taken. Total edits (total incorrect) is the sum of all (incorrect) edits made by the expert over four rounds of editing. Percentage wrong was calculated by dividing total incorrect by total edited. Cumulative impact on the customer earnings, or impact, was calculated as the customer payoff generated by the expert's behavior over all four rounds. As subjects were given the opportunity to leave the experiment early, total time taken is the amount of time the experts spent editing the essays across all four rounds.

Table 2 provides a summary of the actions taken in the different treatments. As discussed in Bejarano et al. (2016), experts preferred either *fee-for-service* or *salary* over *capitation*. Those subjects who self-selected *fee-for-service* provided significantly more edits than those randomly assigned, resulting in more earnings for themselves and less help for their customers. The observed behavior between the randomly assigned *salary* treatment and those who self-selected *salary* did not significantly differ.

**Table 2:** **Actions summary by treatment**.

**Variable**	**Randomly assigned**			**Selection**		
	**Fee-for-service**	**Salary**	**Capitation**	**Fee-for-service**	**Salary**	**Capitation**
Total edited	175.8	81.7	89.2	250.3	85.8	73.8
Total wrong	98.1	7.6	17.6	180.6	13.8	7.0
Total correct	77.8	74.1	71.5	69.7	72.0	66.8
Percentage correct	62%	90%	83%	40%	85%	89%
Cumulative impact	6.8	10.8	9.8	1.1	10.4	9.7
Total earnings	$35	$25	$21	$51	$25	$23
Number of subjects	39	41	25	49	70	4

We begin with a correlational analysis of the relationship between subjects' actions and CPCIS measures. The correlation analysis only captures the way in which actions correlated with specific individual's characteristics. In the second part of this section, we report the results of a cluster analysis that groups subjects acting in similar ways. These clusters were most salient when subjects could self-select into one of the payment schemes. We analyze whether particular subjects' behavior or action-strategy types are revealed by actions in the experiment, and whether we observe differences across types in the *self-selected* treatment. Finally, we analyze how the observed relationships between experimental actions and CPCIS measures relate to our theoretical hypotheses.

### Correlation analysis

We start this section by exploring the individual characteristics across the six experimental subject types: self-selected and three randomly assigned into either *fee-for-service, capitation*, or *salary* types. When comparing across experimental subject types, we do not expect to see much difference between individual characteristics of those subjects that were randomly assigned individuals to the different payment schemes, because they were randomly selected from the general subject population. In contrast, we would expect to see differences in the individual characteristics of those that self-selected different payment schemes.

We proceed as follows: First, we study the correlation between experimental actions and individual characteristics for all those subjects for whom we have the CPCIS data (A summary of each of the CPCIS data measure can be found in the Appendix). This analysis, which includes the pooled set of randomly assigned and self-selected individuals, should reveal if *ceteris paribus* measures within a characteristic category are strongly correlated with actions in a particular way. Second, we use the fact that self-selecting into different payment schemes might reveal something about a subject's type to better understand behavior. Here, we analyze the correlation between each one of the payment schemes disaggregated by self-selection and randomly assigned with each of the individual characteristic measures in the CPCIS data. In both cases, we estimated the Spearman correlation coefficient^3^ and test significance correcting for the multiple hypothesis effects via the Bonferroni adjustment.

In the analysis of the pooled set of subjects, there are two main findings: First and not surprisingly, variables within a characteristic category are typically highly correlated with one another. Second, we did not find any significant correlation between any of the preference measures and subject actions in the experimental treatments. The lack of correlation is consistent with our predictions of individual preferences but surprising for those measures of strategic preferences, which were hypothesized to play a role in behavior in our primary experiment.

One exception is the correlation between all BFMS variables, measures of strategic preference, and action variables in our primary experiments. Particularly, we observed that when evaluating the correlation between the pooled data, i.e., all subjects in all treatments, selfishness correlates positively with total edited (rs = 0. 185, *p* < 0.10). Furthermore, in all three cases, the three variables, egalitarianism, altruism and selfishness, have significant positive correlation with the amount experts earned with rs = 0.158, rs = 0.221, and rs = 0.179, and *p* < 0.10, respectively,. In contrast, altruism is not correlated with the number of wrong edits or its percentage. Furthermore, both egalitarianism and selfishness have a positive correlation with the number of wrong edits (and its percentage) with these respective statistics, rs = 90.1779, rs = 0.214, and *p* < 0.05 in both cases. Accounting for self-selection in general or self-selection into a particular payment scheme, all these correlations hold their significance except the correlation between the number of total edits, which now is not statistically significantly related to egalitarianism.

We found CRT measures correlated with total earnings in two dimensions: The number of correct CRT answers is positively correlated with total earnings (r_s_ = 0.2348, r_s_ = 0.3030, *p* < 0.05), and CRT impulsiveness is negatively correlated with total earnings (r_s_ = −0.2270, *p* < 0.10). This result is consistent with the findings of Corgnet et al. (2015) given that CRT relates to how compulsive/deliberative subjects are. However, these results should not be generalized since these traits could affect both the self-selection and the actions taken by subjects after this choice. Therefore, the outcome could be either driven by the self-selected portion of the subjects or not.

The lack of significant correlation between most of our measures of individual characteristics and subject actions conflicts with the theoretical hypotheses that we discussed in the previous section. *None* of the preference measures were correlated with any of the experimental action variables. Several explanations for this result are feasible. One possible explanation for the lack of correlations is that the CPCIS instrument and the primary experiment were conducted at different times by different researchers. This might imply that subjects are less likely to act in a manner consistent with the behavior characterized by their responses to the CPCIS tasks while performing in the primary experiment. Differences in the timing and circumstances of the CPCIS tasks and the primary experiments imply that their behavior in the primary experiment is less likely to reflect any implicit experimenter demand effect.

We continue our analysis by examining only correlations among those who self-selected the same treatment. This is an important step in our analysis, as the act of choosing a treatment might reveal differences in individual characteristics. To analyze this possibility, we break down the correlation analysis into two steps. First, we conduct the same correlation analysis as above but only for those subjects in the *self-selection* treatment.

Not surprisingly, there is no correlation between experimental actions and the individual characteristic measures of the CPCIS for the subjects that self-selected the two most popular payment schemes^4^. The next step in our analysis is to break down the correlation analysis, controlling for self-selection into a particular payment scheme, *salary* or *fee-for-service*.

The analysis of correlation between subjects that self-selected a similar moral dilemma presents two main findings. First, almost all the finding of the analysis of the pooled set of a subject's data persists. This means that those characteristics that were not found significantly correlated persisted and presented a lack of relationship with actions and were still not correlated when disaggregating by payment scheme. In contrast, we found that the selection choice may work as a screening device of subjects with different values for those that were found significant for all the self-selected subjects. This is reflected by the fact that accounting for the particular payment schemes eliminates the significance for those relationships that were significant for the pooled set of subjects into both payment schemes. This result holds for all the correlations between actions and individual characteristics reflected by variables such as egalitarianism, altruism and selfishness, as well as CRT correct and CRT. This result could be explained if values for these variables and actions are similar among those that self-selected salary but very different for those that self-selected fee for service.

### Cluster analysis

The results of the previous section lead us to believe that there may be different types of experimental subjects. More specifically, we argue that the inconsistencies in correlations between measures of individual characteristics and observed actions are due to the fact that in our primary experiment multiple characteristics, i.e., cognitive capabilities, individual preferences, social preferences and personality traits, might affect behavior. That is, given the moral dilemma and real effort features of our primary experiment, we expect that certain individual characteristics will pull the subject's behavior in opposite directions. For example, experts with high measures of intelligence would be more likely to provide better outcomes for their customers, whereas low levels of altruism imply worse outcomes for their customers. Therefore, a subject's the combination of the individual characteristics each subject possesses may have uncertain implications for theoretical predictions.

For this reason, we next explore if expert actions reveal behavioral types and whether behavioral groups correspond to differences in preference, cognitive, and demographic characteristics. To do this, we use cluster analysis to build behavioral groups from the actions of subjects in the selection treatment of our primary experiment.

Clusters (behavioral groups) are based on a subject's actions. Specifically, behavioral groups are created using the action variables “total edits” and “total incorrect edits.” Cluster analysis based on these two variables allows us to distinguish between subjects who edited a lot with a high percentage of incorrect edits (the Demons) and subjects who edited sparsely with a high percentage of incorrect edits, as well as those who edited few with a high percentage of correct edits (the Angels).

Behavioral groups were created using the *k*-mean algorithm with Euclidian distances. We clustered on values of *k* from 2 to 6 and maximized the Calinski and Harabasz (CH) pseudo f-statistics to find the optimal clustering (Caliński and Harabasz, 1974). In order to control for the robustness of the *k*-mean algorithm, we ran it in a loop with 50 repetitions for each value of *k*. From these repetitions, we selected the cluster with the highest CH pseudo F-statistic for each value of *k*. Then, comparing across the *k* values, we selected the clustering with the highest CH pseudo f-statistic.

#### Behavioral groups

**Figure 1:**
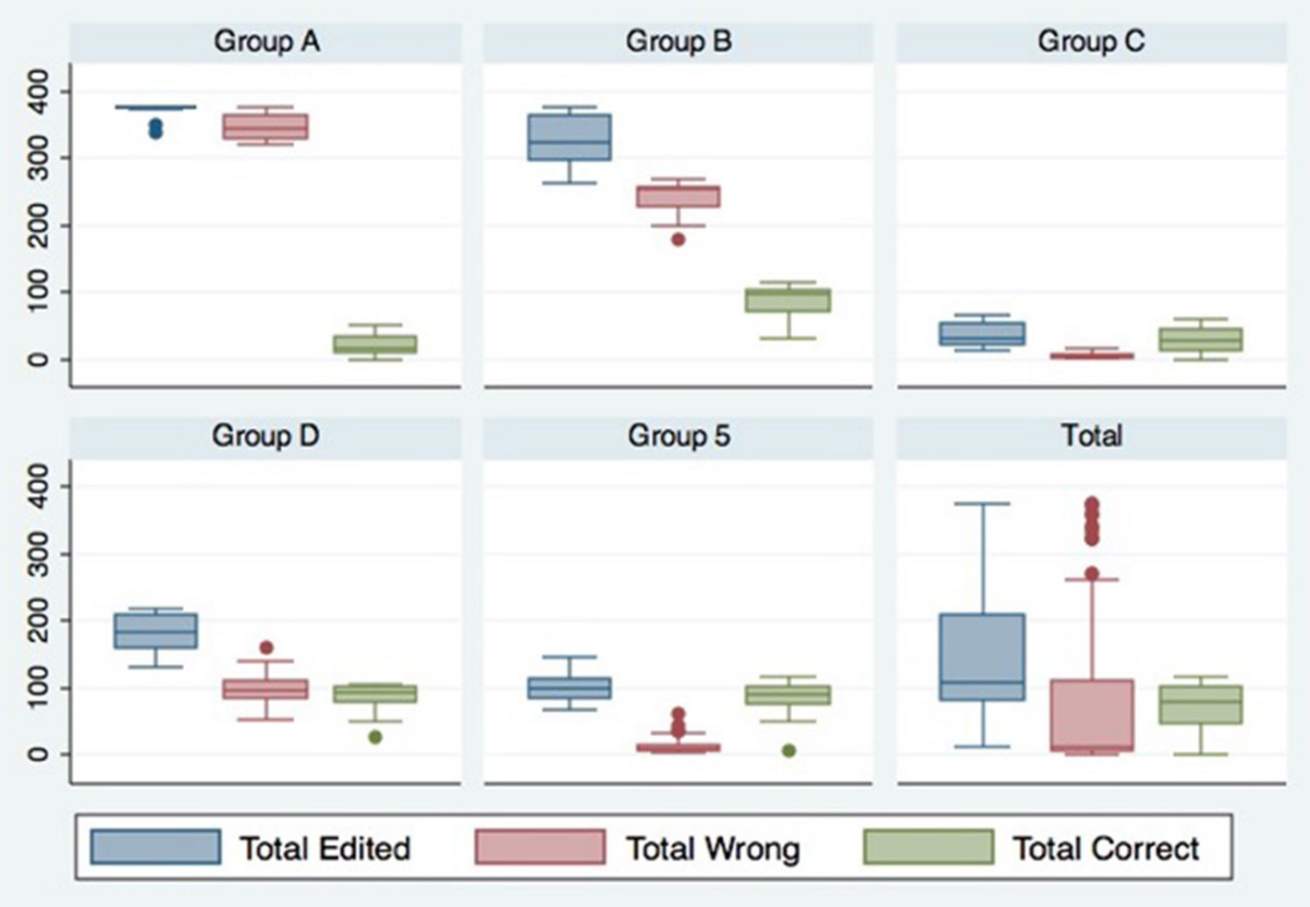
**K mean cluster analysis**.

Figure 1 and Table 3 provide summaries of the cluster groupings. Table 3 summarizes the experimental actions taken by the typical member of the five behavioral groups created by our cluster analysis. The results displayed in Table 3 reveal three things. First, they reveal that various subjects in our primary experiment behaved in very different ways. Second, the significant differences on actions across behavioral groups imply that our cluster methodology identified different types of subjects. Lastly, a large part of subject behavior is captured by the subjects' choices of payment scheme. The payment scheme selection action completely and consistently separates the five groups into two subsets, {A, B, D} and {C, E}. No significant differences exist between any pair of groups from within either subset, but significant differences do exist between any pair of groups across subsets.

**Table 3:** **Actions summary by group**.

**Variable**	**Group A**	**Group B**	**Group C**	**Group D**	**Group E**
Total edited	370	328	37	182	101
Total wrong	347	241	6	99	13
Total correct	23	87	31	83	88
Percentage correct	6%	26%	80%	46%	87%
Cumulative impact	−14	2	5	7	12
Total earnings	$74	$63	$24	$36	$25
Percentage *fee-for-service*	100%	93%	5%	90%	21%
Number of subjects	13	14	19	10	63

However, payment choice does not capture all the dimensions of subject behavior. Figure 1 reveals that even for those groups with a large percentage of subjects choosing the *fee-for-service* payment scheme (Groups A, B, and D), behavior varied significantly. And although a much smaller percentage of subjects chose the *fee-for-service* payment scheme, Groups C and E also displayed dissimilar behavior in other dimensions. For example, although Group E has a large number of subjects choosing *salary* rather than the *fee -for-service*, the *fee -for-service* subjects of Group E (the Angels) behaved very different than *fee-for-service* subjects in Groups A, B and D and, in particular, most different from those in Group A (the Demons).

The experimental actions from our primary experiment show strong support for the existence of behavioral types as revealed in Table 3 and Figure 1. In the spirit of the ongoing claims in various fields of behavioral science, we seek to determine whether the differences in primary experimental behavior relate to individual characteristics that may be captured independently by the CPCIS database. Understanding this question is of great importance to experimental research.

In order to test the hypotheses that there are no differences in the individual characteristics of students who have been clustered into different groups, we perform a binary comparison of the aggregate experimental actions taken by subjects in each pair of groups for each CPCIS characteristic. The complete results for the Two Sample Fligner–Policello Rank Test are displayed in **Table 5**. Table 4 provides a summary of these results by reporting the count of the number of CPCIS characteristics in which each pair of behavioral groups differed significantly.

**Table 4:** **Summary of FPRANK comparisons across groups and individual preferences**.

	**Group A**	**Group B**	**Group C**	**Group D**	**Group E**
Group A	–	6	5	4	3
Group B	6	–	7	5	7
Group C	5	7	–	6	4
Group D	4	5	6	–	2
Group E	3	7	4	2	–
Totals	18	25	22	17	16
% of Total	13%	18%	16%	13%	12%

Although, all the groups were formed by Chapman students, each group displayed at least 2 and up to 9 significant differences in the characteristics of its membership. There were 26 characteristic differences that reinforced the basic subdivision ({A, B, D}, {C, E}) that was revealed by payment selection, but there were 23 characteristic differences between groups within the same subset; this allows us to differentiate between groups that have a similar predilection for payment scheme.

Table 5 displays the differences among those individual characteristics for each group. For each variable in the CPCIS, we provide the information of the mean at the group level and the number of subjects in the group. We also rank them from the highest (1) to the lowest (5) value and the sign for those differences that were statistically significant according to the results of the binary Two Sample Fligner–Policello Rank Test. We next describe how the results in Table 5 relate to the theoretical implications discussed in Section Theoretical Relationship among CPCIS Variables.

**Table 5:** **Summary statistics of action and individual characteristics**.

	**Group A**			**Group B**			**Group C**			**Group D**			**Group E**		
**Variable**	**Mean**	**Ranking**		**Mean**	**Ranking**		**Mean**	**Ranking**		**Mean**	**Ranking**		**Mean**	**Ranking**	
**INDIVIDUAL PREFERENCES**															
Risk aversion	4.00	3	–	4.15	2	>3	4.00	3	<2	3.90	4	–	4.18	1	–
Loss aversion	3.38	3	–	3.15	5	<1	3.28	4	–	3.90	1	>5	3.46	2	–
Time preference	6.00	2	–	6.23	1	–	4.94	5	–	5.30	4	–	5.74	3	–
**STRATEGIC PREFERENCES**															
**Trust**															
Trust sent	7.69	2	–	8.46	1	–	6.11	3	–	6.00	4	–	7.37	3	–
Trust return	13.08	1	>4, 3	10.38	4	<1	13.06	2	>3	9.00	3	<1, 2	12.46	3	–
**Ultimatum**															
Offer	4.85	5	–	5.69	2	–	5.11	4	–	5.70	1	–	5.44	3	–
First accepted offer	3.92	1	–	3.62	4	–	3.67	3	–	3.80	2	–	3.49	5	–
Advantageous offers	0.00	4	–	0.31	2	–	0.50	1	–	0.50	1	–	0.05	3	–
**Dictator**															
Sent	4.31	4	<2	5.62	1	–	5.44	2	>1, 3	4.30	5	–	4.70	3	<1
**Prisoners' dilemma**															
Cooperative action	1.54	1	>4	1.23	4	<1	1.50	2		1.50	2	–	1.39	3	–
**Bartling**															
Egalitarianism	3.54	1	>5	3.07	4	–	3.21	2	>5	2.70	5	<1, 2	3.11	3	–
Altruism	0.92	5	–	1.50	1	–	1.26	3	–	1.40	2	–	1.17	4	–
Selfishness	1.54	2	>5	1.00	5	<2, 1	1.21	3	<1	1.90	1	>5, 3, 4	1.14	4	<1
**INTELLIGENCE**															
**Psychology**															
Raven	13.69	1	–	13.15	3	–	12.17	4	–	12.60	5	–	13.16	2	–
CRT	3.23	2	–	4.08	1	>5	2.67	5	<1	3.10	4	–	3.18	3	–
Wonderlic	19.77	3	–	20.23	1	>5	18.94	5	<1, 2	20.20	2	>5	19.54	4	–
**Numeracy**															
Adding task (Incentivized)	15.38	4	–	17.54	1	>5	14.78	5	<1	16.60	2	–	16.05	3	–
Adding task (Not Incentivized)	13.85	4	<1	17.08	1	>4, 5	12.83	5	<1, 2, 3	16.80	2	>5	15.21	3	>5
**Academic**															
SAT	5.15	4	–	5.38	2	>5	5.29	3	–	5.40	1	–	4.96	5	<2
ACT	6.42	1	–	6.23	2	–	5.82	5	–	5.89	4	–	6.00	3	–
GPA	3.61	2	>5	3.72	1	>5, 3	3.41	5	<2, 1, 3	3.45	4	–	3.56	3	<1, >5
**Social**															
Theory of mind	27.69	1	–	27.30	2	–	26.41	5	–	26.50	4	–	27.02	3	–
**PERSONALITY-BIG 5**															
Openness	36.54	4	–	39.08	1	–	36.94	3	–	36.40	5	–	37.28	2	–
Conscientiousness	30.77	5	–	32.23	2	–	32.06	3	–	31.10	4	–	32.51	1	–
Extroversion	28.08	1	>4	26.85	3	–	27.28	2	–	26.60	4	<1	25.86	5	–
Agreeableness	31.77	4	<3, 2, 1	31.54	5	<1, 2	33.72	3	>4	35.20	1	>4, 5	34.49	2	>4, 5
Neuroticism	25.85	1	>4, 5	24.08	2	–	21.50	4	<1	20.00	5	<1, 3	23.61	3	>5
**DEMOGRAPHICS**															
Age	18.85	4	–	18.62	5	<1	18.89	3	–	18.90	2	–	19.23	1	>5
Gender	1.54	2	–	1.43	5	–	1.53	3	–	1.60	1	–	1.44	4	
Volunteer hours	1.15	5	<1	1.67	1	>3, 4, 5	1.47	3	<1	1.22	4	<1	1.49	2	
Work hours	1.46	5	<1	2.25	1	>3, 4, 5	1.76	3	<1	1.89	2	–	1.60	4	<1
Number of siblings	1.23	4	<1	1.15	5	–	1.56	2	–	1.50	3	<1	1.91	1	>3, 4
Older siblings	0.77	4	–	0.46	5	<3	0.83	3	>5, <1	1.10	1	>**3**	0.88	2	–
Religiosity	1.67	3	–	1.54	4	<1	1.41	5	<2	1.90	2	>5	2.04	1	>4

*Subjects who chose the capitation payment were not able to be clustered into a group. Their data is not summarized here*.

*Significance in ranking reported by <or > signs next to ranking*.

#### Individual preferences

In contrast to the correlation analysis where no measures of individual preferences were significantly different, risk aversion, and loss aversion were each significantly different between two groups (Groups B > C and Group D > Group B, respectively). This is surprising as experts' risk aversion and loss aversion profiles should not affect their behavior as the subjects are in control of their actions and thereby, their earnings. However, these results cannot be rationalized by either a non-egocentric egocentric view of preferences over other's risk and loss (Hsee and Weber, 1997). If we consider each action as a choice between an uncertain outcome (i.e., edit is potentially right or wrong) and a certain outcome (i.e., no edit means no risk) for their counterpart, the number of edits conducted would reflect one's risk aversion. However, Group C behaved more conservatively in editing than Group B, whereas Group C is less risk averse than Group B. In contrast, Group B had lower levels of loss aversion than Group D and conducted significantly more edits than Group D. This demonstrates Group B's willingness to act carelessly in decisions that negatively impact others more so than the behavior of Group D. These results provide a first indication of how difficult it is to relate measures of individual preference to behavior in a real-effort moral dilemma.

#### Strategic preference

In contrast to the correlation analysis, more measures of strategic preferences were found to be significantly different. First, we predicted that subjects with higher levels of reciprocity would act more benevolently than others in our primary task; that is, these subjects would provide a higher cumulative impact (income) for their customers. However, we found that when comparing the actions in the Trust Game (reciprocity), groups with high levels of reciprocity were less benevolent to their customers. For example, groups that provided a large number of incorrect edits, such as Group A (the Demons), or relatively few edits, such as Group C, had higher rankings of reciprocity, i.e., returned more money on the trust game. However, it is important to note that in the Trust Game, reciprocity from the recipient is conditional whereas, in our experiment, expert actions toward customers are not. In our primary experiment, the benefits experts confer to their customers do not affect their own earnings. This key distinction may explain the unexpected behavior.

BFMS measures of egalitarianism, selfishness or altruism also partly contradict our theoretical predictions. Groups A and C had significantly larger measures of egalitarianism in the BFMS relative to the other groups; however, in our experiment Group A's actions reflect those of *homo economicus* and Group C's reflected those of an egalitarian. Group C has the lowest overall personal earnings and the 3rd highest cumulative impact for their customer. A similar relationship appears with measures of selfishness reported by the BFMS. Groups A and C are among those with higher levels of selfishness. The inconsistency in behavior and similarity of BFMS measures in these two Groups leads us to question the usual interpretation of the BFMS measure.

#### Intelligence

We found that 6 of our 9 measures of intelligence differed across groups. In contrast to the simple correlation analysis, the different behavioral groups were not drawn from the same population with respect to the CRT and Wonderlic test results. For the CRT we found that Group B, the group with the highest CRT values, also behaved in a way that could be described as mildly altruistic and selfish. Group B mostly opted for *fee-for-service* and conducted a large number of edits, thereby increasing their earnings. However, relative to Group A, who also provided a large number of edits, Group B provided more accurate edits. This behavior we characterize as mildly altruistic and selfish, and it is consistent with our theoretical predictions. Similar results were observed by Corgnet et al. (2015). Corgnet et al. observed in their experiments that individuals with high CRT scores behave in a more altruistic way.

Our final measure of intelligence that had significant differences across groups is by numeracy both in an incentivized task and not incentivized. In our theoretical predictions, we argue that there is no relationship between numeracy and the task in our primary experiment. However, upon reflection, statistically significant differences on the not incentivized Adding Task would not contradict our predictions. Notice, that this CPCIS task measures more than numeracy skills, as subjects are not presented with any monetary incentive to add correctly. Therefore, the correct additions performed in this task is also a measure of intrinsic motivation. Hence, finding that those who groups with the highest number of correct edits (Groups B, D, and E) also have the highest scores on the not incentivized Adding Task is as one would expect.

#### Personality

Table 5 reveals that the behavioral groups differ with regard to at least three personality measures (Extroversion, Agreeableness, and Neuroticism). Two measures, however, Openness and Conscientiousness, are not statistically different among groups. The finding that Conscientiousness does not differ amongst groups regardless of choice of payment contradicts our theoretical discussion. However, we do find two supporting results. First, Group A (the Demons) has higher levels of extroversion. This group is only composed of subjects that chose *fee-for-service*; it conducted the most edits on average and had the lowest percentage of correct edits, all of which are consistent with an extrovert's attitude toward rewards. Second, Groups D and E showed the highest values of Agreeableness. These two groups also had higher cumulative impact (the return of dollars to customers as a result of their actions). This result is consistent with the compassionated attitude associated with this trait. Finally, we also found that Neuroticism differs significantly among groups. Particularly, Groups A, B, and D, the three groups with the largest numbers of edits, presented higher values of Neuroticism than the Groups with lower numbers of edits, Groups C and E.

#### Demographics

Several demographic characteristics presented significant differences amongst groups. Of particular interest to our analysis are self-reported numbers of volunteer and work hours. Again, Group B ranked the highest for these two variables. We have already described the behavior of Group B as mildly altruistic and selfish, so it is encouraging that the results are consistent with our previous finding.

## Discussion

In general, understanding how individual characteristics influence behavior is a fundamental task of the economist, psychologist, and scientist. While crucial, scientists rarely have independent datasets that combine both an individual subject's characteristics and behavior (Caplan, 2003). In this article, we leverage a uniquely large dataset containing the individual characteristics of a subset of our experimental subjects to shed light on the relationship between subject's individual characteristics and their behavior in a real-effort moral dilemma with self-selection by payment scheme. Due to the unique nature of our primary experiment, we use two statistical approaches, correlation analysis and cluster analysis, to better understand these dynamics. Different scholars collected our two datasets at different times for different reasons. This allowed us to avoid issues associated with a sequence of primary and secondary experiments conducted by the same experimental team and setting. The following points summarize our results.

First, there is no clear majority of individual characteristics that correlate with behavior in our primary experiment. A set of a few, but interesting, significant correlation relationships were found across experimental actions. We found that no measure of individual preference, i.e., time discounting, risk and loss aversion, was significant. Furthermore, our measures of strategic preferences, which include variables such as Trust, Trustworthiness, and Altruism captured from implementation of canonical Trust and Ultimatum Games also failed to show any significant correlation with actions in a real-effort moral dilemma. These results highlight the importance of conducting reliability tests for simple statistical analyses exploring these social preferences (Charness and Rabin, 2002). Other measures of social preferences, such as those derived from Bartling et al. (2009), were significantly correlated with actions, but the results of these correlations were contradictory to our theoretical predictions.

Measures of intelligence and personality traits also often failed to correlate with observed behavior in our primary experiment. This result is consistent with previous findings (Becker et al., 2012) and presents an additional call to a better development in the study of the relationship between personality traits and economic behavior (Almlund et al., 2011; Rustichini et al., 2012). Hence, there is a need for replication of this investigation to develop better theoretical models (Benjamin et al., 2013).

There are several arguments that may justify these inconsistencies. First, ordering of tasks has been shown to impact outcomes in experiments. For instance, Healy et al. (2016) demonstrates that by going from a single shot of a game to a repeated game, subjects' payment functions change and thereby, so do behaviors. Similarly, implementing a sequence of tasks, primary and secondary, or a battery of tasks and surveys, as with the CPCIS, could induce different behavior through wealth and portfolio effects. Here, we analyze the correlation between a single task (our primary experiment) and a battery of instruments (the CPCIS), collected on separate occasions. Therefore, behavior in our primary experiment should be less affected by the behavior of the CPCIS than if both datasets had been collected in the same session, producing less consistent correlations than otherwise.

Secondly, it is possible that the joint implementation of tasks in the CPCIS dataset generate spurious correlations. These spurious correlations could be generated from the bundling of experimental tasks, experimenter demand effects (Zizzo, 2010), or idiosyncratic effects of experimenter teams or their lab set up. For instance, researchers often only conduct secondary experiments that they believe will reveal something about their subjects. This potentially introduces an experimenter demand effect instead of the desired elicitation of additional characteristics, resulting in spurious correlations. We argue that the difficulty of finding significant correlations in our analysis, when both of the datasets were collected by separate research teams, is a call for attention to the interpretation of correlations found between primary experiments and secondary measures that are jointly collected. Furthermore, these findings open several research questions regarding how to implement and analyze the results of several experimental tasks, which *a priori* are correlated.

Following the correlation analysis, we found that actions in a primary experiment could be used to categorize subjects into groups based on their observed actions using cluster analysis (i.e., behavioral groups). Furthermore, because of the availability of the CPCIS data we could proceed one step further than several experiments, which have already utilized cluster analysis with the investigation of individual characteristics (Houser et al., 2004; Rong and Houser, 2015).

The cluster analysis reveals that individual characteristics are a distinguishing factor across behavioral groups. Individual measures of preferences (Risk and Loss aversion), strategic preferences (Trust Game, Dictator Game, Prisoners' Dilemman, and Bartling) and Intelligence (CRT, Wonderlic, and numeracy) all varied across behavioral groups. However, like the correlation analysis, the results often contradicted our theoretical predictions. Regardless, it is important to note that these behavioral groups revealed systematic differences in behavior regardless of inconsistencies with theoretical predictions. That is, due to the tension between some of our theoretical analyses of the influence of personal characteristics on behavior in our moral dilemma and the observed behavior, either our theory or our measures are still far from perfect.

Our results suggest that the effects of psychological, cognitive, and demographic differences on behavior in experiments are more complex than those implied by *ceteris paribus* hypothesis. Subjects are endowed with mixtures of individual characteristics that could present contradictory theoretical interpretations. Despite this difficulty, characteristics of subjects that chose and act similarly (i.e., belong to the same behavioral group) are more likely to be similar between each other and different from those that chose and act differently in individual characteristics. This finding could not be detected using a simple correlation analysis. We believe that the results of our analysis shed light on the strength of the links between individual characteristics, behavior in simple strategic games, behavior in real-effort moral dilemmas.

## Author contributions

Each author contributed to the design, implementation, analysis, and writing of the document.

### Conflict of interest statement

The authors declare that the research was conducted in the absence of any commercial or financial relationships that could be construed as a potential conflict of interest.

## Footnotes

^1^Subjects who completed their task before the time was up were asked to raise their hand and were then given a short survey to complete silently. Once finished with the survey, subjects quietly exited the room and were paid outside of the laboratory. We found no session effects.

^2^It is not within the scope of this article to describe each task in the CPCIS in detail. Most of the tasks included in the CPCIS have been used in several experiments. In this case, we make an exception for the BFMS, assuming that it is not as well-known as the other tasks. Still, we encourage the reader to read Bartling et al. (2009) and Corgnet et al. (2015) for more detailed descriptions of this type of instrument.

^3^The Spearman's rho is a nonparametric measure of rank correlation. The assumption of monotonicity in Spearman's rho test is satisfied.

^4^Total time taken showed a positive correlation with self-reported GPA (r_s_ = 0.1981, *p* < 0.10).

## Appendix

**Table A1:** **CPCIS Taxonomy**.

**Measure**	**Method–Test (Reference)**	**Related elicited characteristic**
**INDIVIDUAL PREFERENCES**		
		Preferences over:
Risk aversion	Multiple listing method (Andersen et al., 2006)	Risk over lotteries
Loss aversion	Multiple listing method (Andersen et al., 2006)	Lotteries with losses
Time	Multiple listing method (Andersen et al., 2006)	Temporarily based payments
**STRATEGIC PREFERENCES**		
**Trust**	Trust Game (Berg et al., 1995)	
Trust sent	Trustor can sent only 0 or 10	Trust
Trust SM return	Trustee respond to each possibility (Strategy Method SM)	Reciprocity
**Ultimatum**	Ultimatum (Güth et al., 1982)	
Offer	Strategy method, first player can send any even number between (0–20, 11 choices)	Altruism
First accepted offer	When playing as a second player, player can reject any proposal	Fairness
Number of advantageous offers rejected		Fairness
**Dictator game**	Dictator game (Kahneman et al., 1986)	
	Strategy method, player can send any even number between (0–20, 11 choices)	
**Egalitarianism, Altruism, and Selfishness**	Bartling, Fehr, Marechal, and Schunk (BFMS) Task from Bartling et al. (2009)	
Egalitarianism	Zero to six choices of an equitative alternative over non-equitative	Preferences for equitatives distribution
Altruism	Zero to three choices of a detrimental alternative over equitative alternative	Preferences for distribution that benefit others
Selfishness	Zero to three choices of a beneficial alternative over equitative alternative	Preferences for distribution that benefit herself
**INTELLIGENCE**		
**Pyschology**		
Raven	Reduced version of the Raven Test in this case subjects have to choose only 18 questions	Fluid intelligence
Cognitive reflection test (CRT)	Extended version of the CRT described by Toplak et al. (2011)	Reflection and impulsiveness
Wonderlic	Reduced version of the Wonderlic Personnel Test, 24 questions with a maximum of 6 min	
**Numeracy**		
Adding task correct (incentivized)	Individual has to add 10 sequences of summations and its pay for each correct addition	Numeracy capabilities with extrinsic motivation
Adding task (no incentivized)	Similar to the incentivized task but individuals are not paid by correctness	
**Academic**		
SAT	Self-reported	
ACT	Self-reported	
GPA	Self-reported	
**Other**		
The reading the mind in the eyes test	A sample test in which individuals were requested to guess the most likely emotion of 36 pictures of eyes	Theory of mind
**PERSONALITY—BIG FIVE**		
Openness	Big five questionnaire with 44 items, John et al., 2008	Include traits of appreciation for unusual ideas, curiosity, and variety of experience
Conscientiousness	Big five questionnaire with 44 items, John et al., 2008	Tendency to be organized, disciplined, dependable, and to prefer planned behavior
Extraversion	Big five questionnaire with 44 items, John et al., 2008	Tendency to seek stimulation by the company of others, to be talkative, energetic, and assertive
Agreeableness	Big five questionnaire with 44 items, John et al., 2008	Tendency to be sympathetic, compassionate and cooperative, kind, and affectionated
Neuroticism	Big five questionnaire with 44 items, John et al., 2008	Tendency to be moody, and to experience easily emotions such as anger, anxiety, and depression
**DEMOGRAPHICS**		
Age	Self-reported	Numeric
Gender	Self-reported	Male or female
Volunteer hours	Self-reported	Range of number of hours allocated to voluntary work or N/A
Work hours	Self-reported	Range of hours allocated to remunerated work
Number of sibling	Self-reported	Range of number of hours allocated to remunerated work or NA
Older sibling	Self-reported	Number of siblings older than the subject
Religiosity	Self-reported	Range of the frequency of service attendance
